# Reference gene selection for cross-species and cross-ploidy level comparisons in *Chrysanthemum* spp.

**DOI:** 10.1038/srep08094

**Published:** 2015-01-28

**Authors:** Haibin Wang, Sumei Chen, Jiafu Jiang, Fei Zhang, Fadi Chen

**Affiliations:** 1 College of Horticulture, Nanjing Agricultural University, Nanjing 210095, China; 2 Jiangsu Province Engineering Lab for Modern Facility Agriculture Technology & Equipment, Nanjing 210095, China

## Abstract

The establishment of a (set of) stably expressed reference gene(s) is required to normalize transcription data. Polyploidy is very common in the plant kingdom, but it is not necessarily the case that a reference gene which works well at the diploid level will also work well at the polyploid level. Here, ten candidate reference genes are compared in the context of gene transcription in the genus *Chrysanthemum*. The robustness of some, but not all, of these was shown to be high across ploidy levels. *MTP* (metalloprotease) and *ACTIN* (actin) were the most stable in diploid and tetraploid *C. nankingense*, while *PSAA* (photosynthesis-related plastid gene representing photosystem I) and *EF-1α* (elongation factor-1*α*) were the most stable in tetraploid and hexaploid *C. zawadskii.* *EF-1α* and *PGK* (phosphoglycerate kinase) was the best combination for the complete set of four taxa. These results suggest that when making cross-species comparison of transcript abundance involving different ploidy levels, care needs to be taken in the selection of reference gene(s).

The measurement of specific transcript abundance, particularly in response to external treatments or to genetic manipulation, is a feature of many plant studies^1^,^2^. A frequently used platform for this purpose is the quantitative real time PCR (qPCR) assay, thanks to its low cost, sensitivity, flexibility and scalability^2^. The accuracy of the platform is heavily dependent on an appropriate choice of reference gene(s) used to normalize transcript abundances. The transcription of an ideal reference gene must be stable both in time and space, and be unaffected by any treatment or genetic manipulation involved^2^,^3^,^4^. A number of genes have been used for this purpose, even though it is clear that some of them are not as stable as is required^5^,^6^. Therefore, it is necessary to select suitable reference genes that are steadily expressed in the relevant experimental conditions before determining the expression pattern of a target gene by qRT-PCR^3^,^7^. The two algorithms geNORM^8^ and NormFinder^9^ have been developed as a means to identify optimal sets of reference gene(s) in specific experimental conditions^10^,^11^,^12^.

Polyploidy is widespread among the angiosperms, so much so that all lineages reflect at least one polyploidization event. Some species, referred to as cryptic polyploids, were long believed to be genuine diploids, but have turned out to harbour duplicated genome segments, so are thought to represent decayed polyploids. Combining two independent genomes within a single nucleus has profound effects on both DNA sequence and the transcriptome, a phenomenon commonly referred to as “genomic shock” which was predicted by McClintock^13^. Research has shown that polyploid formation is typically followed by large-scale changes in gene expression^14^,^15^. Although some reference genes have been shown to be effective over a wide range of developmental and environmental conditions within a species, little attention has been paid to whether the same reference gene(s) can be used in species comparisons in which variation in ploidy level is involved.

In a previous study, we investigated the expression dynamics of 21 selected reference genes in *Arabidopsis* diploid and polyploid species using published transcriptome data to provide a control dataset for gene expression studies of natural variations, in particular *Arabidopsis* tetraploid species^16^. However, suitable reference genes for use in cross-species comparisons within the genus without transcriptome data have never been methodically identified. The genus *Chrysanthemum* harbors species ranging in ploidy from diploid (2*n* = 2*x* = 18) to decaploid (2*n* = 10*x* = 90)^17^,^18^,^19^ and at least three ancient whole genome duplication (WGD) events have occurred during their evolution^20^,^21^. Here we report variation in the transcription of a set of standard reference genes in the leaves of diploid and tetraploid *C. nankingense* and tetraploid and hexaploid *C. zawadskii* by quantitative real-time RT-PCR using geNORM and NormFinder analysis.

## Results

### Performance of the primers

**Figure 1:**
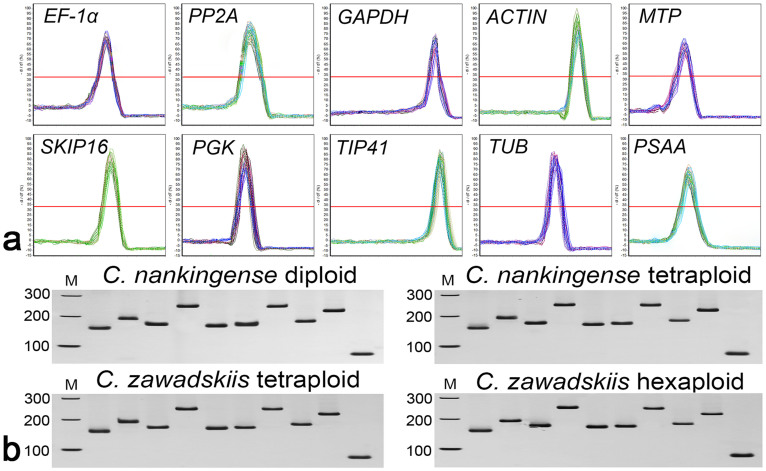
Amplicon dissociation curves and PAGE analysis. (a) Melting curve analysis of the ten reference genes. (b) PAGE analysis of the amplicons. Marker from top to bottom: 300 bp, 200 bp and 100 bp. The samples in each PAGE separation are ordered from left to right as *EF-1α*, *PP2A*, *GAPDH*, *ACTIN*, *MTP*, *SKIP16*, *PGK*, *TIP41*, *TUB* and *PSAA*.

A total of ten genes were selected as candidate reference genes in leaves (the third and fourth true leaves from the shoot apex without any treatment) from diploid (2*x*) and tetraploid (4*x*) *C. nankingense*, and 4*x* and hexaploid (6*x*) *C. zawadskii*. The primer and amplicon characteristics of these ten genes are summarized in Table 1. Melting curve (Fig. 1a) and PAGE (Fig. 1b) analyses showed that each primer pair amplified a single PCR product of the expected size. Analysis using the LinRegPCR program showed that amplification efficiency ranged from 0.8926 to 0.9952, and that the correlation coefficients ranged in value between 0.9922 and 0.9985 (Table 1).

### Transcript abundances

**Figure 2:**
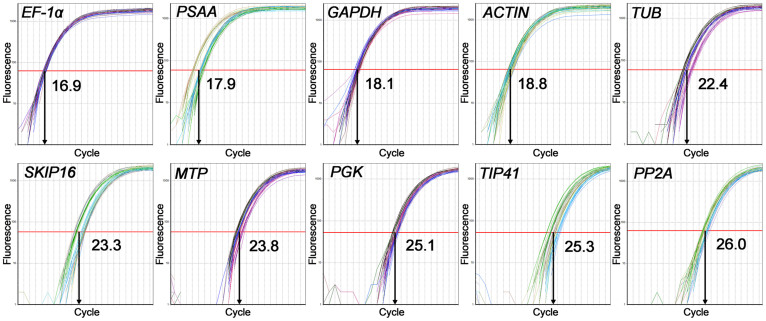
Amplification curves: the X axis represents the PCR cycle number (0–40). The red line represents the threshold fluorescence at which the Ct was determined.

Within a taxon, the Ct value of each candidate gene varied very little among either the four biological replicates or the three technical replicates. These results suggested that the use of different biological replicate did not cause a significant difference. The lowest Ct was 16.8 (4*x* *C. nankingense*, *EF-1α*) and the highest 26.3 (6*x* *C. zawadskii*, *PP2A*). Across all templates, *EF-1α* was the most abundantly transcribed gene, accompanied by the lowest mean Ct (16.9), followed by *PSAA* (17.9), *GAPDH* (18.1), *ACTIN* (18.8) and *TUB* (22.4); *PP2A* was the least abundantly transcribed gene, accompanied by the highest mean Ct (26.0), followed by *TIP41* (25.3), *PGK* (25.1), *MTP* (23.8) and *SKIP16* (23.3) (Fig. 2). However, none of the reference genes were uniformly transcribed in both ploidy forms of either *C. nankingense* or *C. zawadskii*. Therefore, it was necessary to evaluate the reference genes for normalization under the different ploidy level and species.

### Stability of the references genes

**Figure 3:**
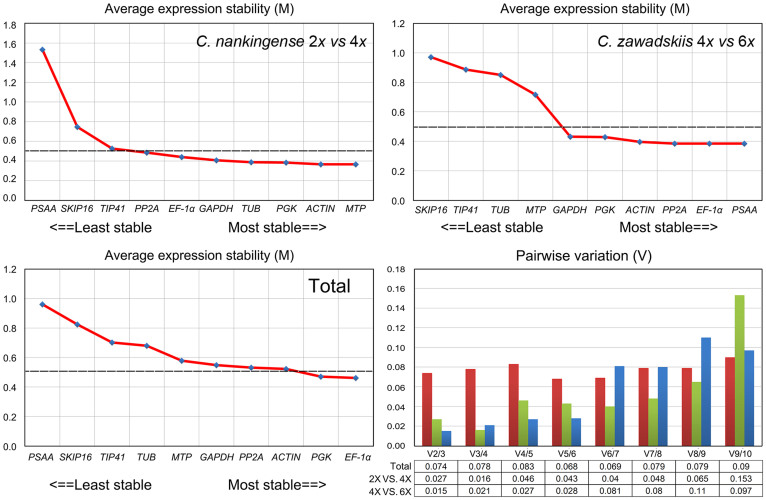
Average expression stability values (M) of the ten reference genes and the determination of the optimal number of reference genes required for normalization. Pairwise variation (*V_n_*_/*n* + 1_) was analyzed between reference genes *n* and *n* + 1 to optimize normalization in the contrasts 2*x* *vs* 4*x* *C. nankingense* (red), 4*x* *vs* 6*x* *C. zawadskii* (green) and between all four taxa (blue).

**Figure 4:**
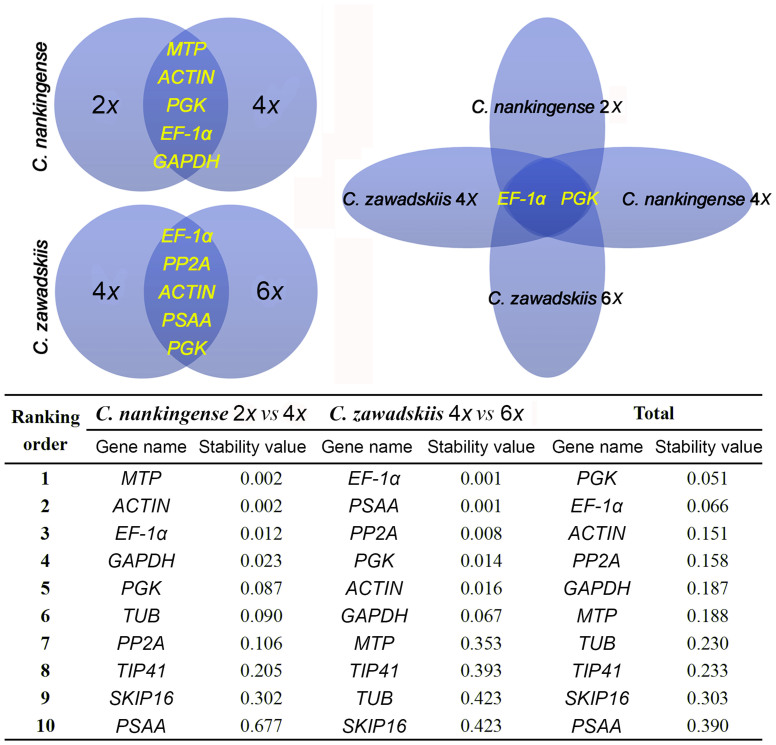
Venn diagram to identify stably transcribed reference genes in *Chrysanthemum* spp., and their stability values calculated using NormFinder.

A geNorm based analysis, carried out to determine which of the reference genes would be most suitable in each of the four taxa, produced the ranking based on M values, as depicted in Fig. 3. On the basis that an M value of <0.5 indicates stability, *MTP*, *ACTIN*, *PGK*, *TUB*, *GAPDH* and *EF-1α* were considered to be stable in the contrast 2*x* *vs* 4*x* *C. nankingense*, while *PSAA*, *EF-1α*, *PP2A*, *ACTIN*, *PGK* and *GAPDH* were all stable for 4*x* *vs* 6*x* *C. zawadskii.* The least stable gene in the comparison 2*x* *vs* 4*x* *C. nankingense* was *PSAA*, even though this gene was stable in the contrast 4*x* *vs* 6*x* *C. zawadskii*. The most stable genes across all four taxa were *EF-1α* and *PGK*, while *PSAA*, *SKIP16* and *TIP41* all performed poorly (Fig. 3). The pairwise variation parameter V calculated by geNorm predicts the optimal number of reference genes required. A V_n_/V_n + 1_ ratio <0.15 is taken to indicate that no additional reference gene is necessary^22^,^23^. Here, the optimal number of genes was two in every case (Fig. 3). NormFinder analysis generated a very similar ranking to the GeNorm output. The combinations *MTP*/*ACTIN*, *EF-1α*/*PSAA* and *EF-1α*/*PGK* were predicted to deliver the most reliable level of normalization (Fig. 4).

**Figure 5:**
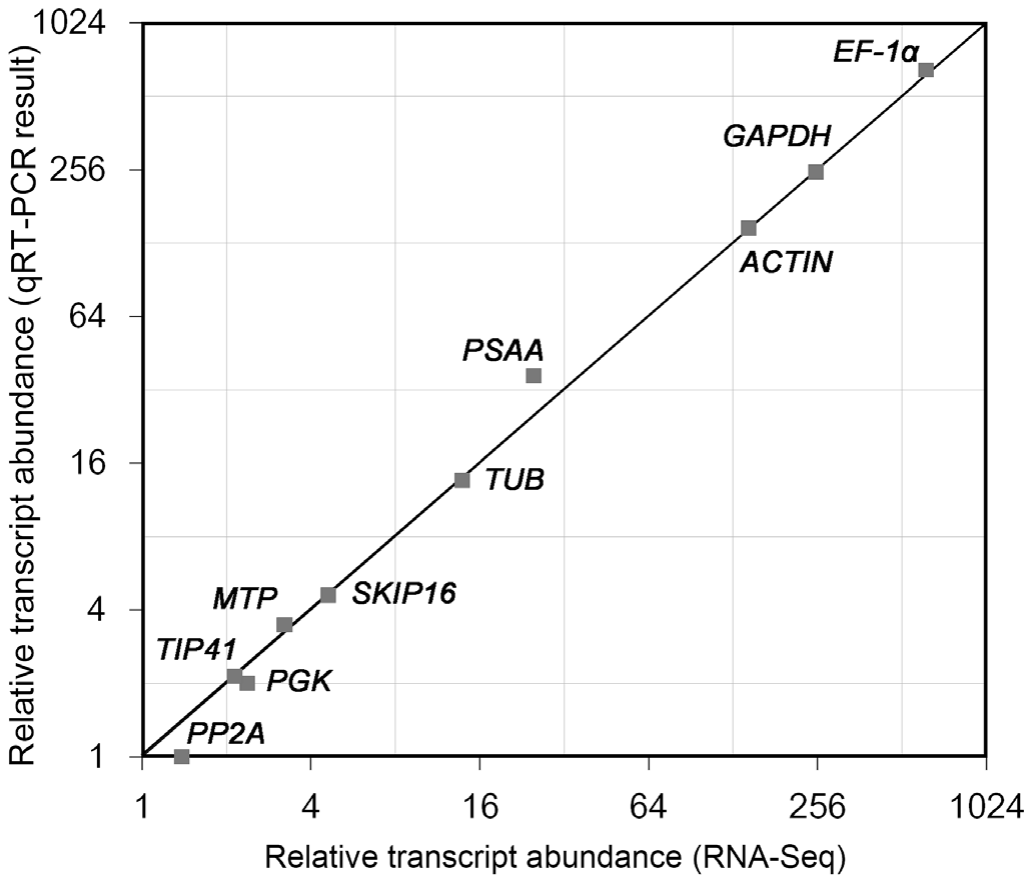
Transcript abundance as estimated from qPCR analysis and from RNA-Seq data. The X axis represents relative transcript abundance from RNA-Seq, and the Y axis the relative transcript abundance estimated from qPCR.

**Figure 6:**
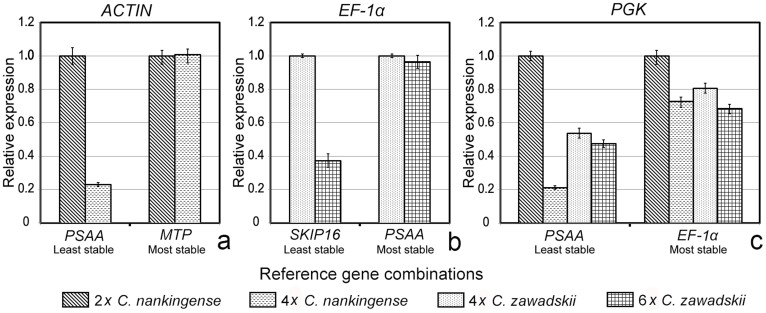
Relative transcript abundances of *ACTIN*, *EF-1α* and *PGK* across species and ploidy levels. Normalization performed using single either stable or unstable reference genes in the contrasts. (a) Relative abundance of *ACTIN* transcript in 2*x* *vs* 4*x* *C. nankingense* (reference genes *PSAA*/*MTP*), (b) relative abundance of *EF-1α* transcript in 4*x* *vs* 6*x* *C. zawadskii* (*SKIP16*/*PSAA*), and (c) relative abundance of *PGK* transcript between all four taxa (*PSAA*/*EF-1α*).

A comparison was made between transcript abundances in 2*x* *C. nankingense* as estimated from qPCR (2^−ΔΔCt^ method^24^) with those derived from RNA-Seq analysis (NCBI SRA accession: SRP049642, be measured by reads per kilobase of exon model per million mapped reads, RPKM^25^). Two methods produced the comparable transcript abundances, especially for *ACTIN*, *EF-1α* and *GAPDH* (Fig. 5). Hence, the Ct value could represent the relative transcript abundance in some extent. Indubitably, the transcript abundance (as well as the Ct) of *ACTIN*, *EF-1α* and *PGK* was similar (the second stable) in the contrasts 2*x* and 4*x* *C. nankingense*, 4*x* and 6*x* *C. zawadskii* and all four taxa, respectively (Fig. 2). To test the importance of the choice of reference gene(s), the relative abundances of *ACTIN*, *EF-1α* and *PGK* transcript were quantified, based on normalization carried out with either a stable or an unstable reference gene. When normalized on the basis of the three most stable reference genes, transcript abundance proved to be stable in the contrasts 2*x* *vs* 4*x* *C. nankingense* (*MTP*), 4*x* *vs* 6*x* *C. zawadskii* (*PSAA*) and all four taxa (*EF-1α*); conversely, when normalized on the basis of the least stable reference genes, this was no longer the case (Fig. 6).

### Application of the qRT-PCR protocol to evaluate the expression of RAD51 gene

**Figure 7:**
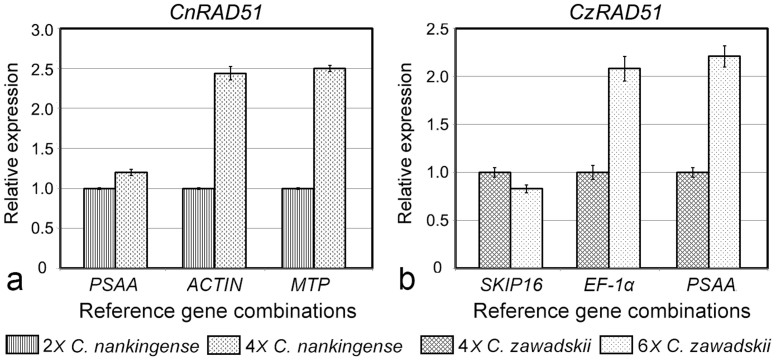
Relative abundance of *RAD51* transcript, estimated from qPCRs based on either stable or unstable reference genes. (a) The contrast 2*x* *vs* 4*x* *C. nankingense*, using as reference genes either *PSAA* or *MTP*/*ACTIN*, (b) the contrast 4*x* *vs* 6*x* *C. zawadskii*, using as reference genes either *SKIP16* or *PSAA*/*EF-1α*.

To demonstrate the usefulness of the validated candidate reference genes, the transcription levels of *RAD51* in both *C. nankingense*, and *C. zawadskii* were estimated using qPCR (the isolation and primer details provided in Supplementary information). Current knowledge of the functions of the RAD51 was known to be a key pathway in cells for the homologous recombination and repair of DNA damage^26^. This gene was chosen because polyploidization events in the genus *Chrysanthemum* are associated with a certain range of DNA repair disorder^27^, which can be expected for a high transcript abundance of *RAD51* in 4*x* *C. nankingense* and 6*x* *C. zawadskii*. However, when normalized using a variety of reference genes, there was a substantial degree of divergence in its estimated relative transcript abundance. Based on the unstable reference genes *PSAA* and *SKIP16*, the level of transcription of *RAD51* was almost the same in 2*x* and 4*x* *C. nankingense* and similarly between 4*x* and 6*x* *C. zawadskii*. In contrast, estimates based on normalization using the most stable reference gene combination (i.e., *ACTIN* and *EF-1α*), suggested that there was a more than two fold difference in transcript abundance in the higher ploidy species (Fig. 7).

## Discussion

### The diversity of reference gene expression in different Chrysanthemum species and ploidy levels

The qPCR platform has been used in many experiments designed to monitor the transcription response to external treatment and/or genetic manipulation^28^. The reference genes used in these experiments have been chosen on the basis that they encode house-keeping proteins which are unlikely to regulate by the treatments imposed. Examples are genes involved in glucose metabolism (*GAPDH* and *PSAA*), in protein degradation (*MTP*), in the regulation of phosphorylation (*PP2A*), in determining cytoskeletal structure (*TUB* and *ACTIN*), in protein synthesis (*EF-1α*) or binding (*SKIP16*); others include genes encoding protein kinase (*PGK*) and the tonoplast intrinsic protein TIP41^4^. Here, the stability of ten widely used reference genes was tested in two ploidy forms of each of two *Chrysanthemum* species. Even though each of these genes is known to be stably transcribed throughout development and under a range of environmental conditions within a species^4^,^29^,^30^,^31^, it became clear that comparisons across species or between ploidy levels within a species would require more than one reference gene (Fig. 3 and Fig. 4). *PSAA* was the most stable single gene in the comparison between 4*x* and 6*x* *C. zawadskii*, but performed poorly in the comparison 2*x* *vs* 4*x* *C. nankingense*. Meanwhile, *EF-1α* and *PGK* were stably transcribed at all three ploidy levels across the two species, which implies that these would represent a good choice of reference gene for inter-species and inter-ploidy level comparisons (Fig. 6 and Fig. 7).

### Possible mechanisms for variation in reference gene stability

Comparisons between different *Arabidopsis thaliana* accessions (and some tetraploid *Arabidopsis* spp.) have shown that the transcription of the genes encoding a pentatricopeptide repeat protein and F-box protein are rather variable, and particularly so in contrasts made between a diploid and a tetraploid accession. *Chrysanthemum* species vary in ploidy level from 2*x* to 10*x*, but large volumes of transcriptomic date have yet to be assembled for these taxa. The present qPCR data demonstrated that the reliability of some genes used for qPCR reference purposes is questionable when they are used in comparisons across species or across ploidy levels (Figs 6 and 7). The data underline the need for care to be taken in the selection of reference genes for specific polyploid combinations.

Microarray and next generation sequencing technology based experiments have delivered plenty of evidence to show that transcription levels vary between species, between ploidy levels and between tissue types^32^,^33^. In some cases, this variation is not revealed by standard qPCR experiments where transcription is normalized using a reference gene(s), although its detection can be improved by spiking with mRNA in proportion to the genomic DNA concentration (for transcripts per cell method)^34^. An alternative approach expresses abundances on a per transcriptome basis after quantifying the size of the transcriptome (after quantifying whole transcriptome size)^35^. These methods will greatly increases the size and complexity of an RT-PCR experiment. A more direct approach, as taken here, is to identify a set of stable reference genes (stable in the sense that their transcripts form a constant proportion of the transcriptome taken across species/ploidy levels). Special emphasis has been concentrated on the possibility of comparison across species and ploidy levels using reference gene(s). Although the reference genes identified here may be less appropriate for experiments involving other species, ploidy levels, tissues or treatments, a rigorous guide for identifying good reference genes has been been followed, in line with the “Minimum Information for publication of Quantitative real-time PCR Experiments” guidelines^36^,^37^.

Polyploidy, in which two or more genomes are combined into a single nucleus, induces a range of both genomic and transcriptomic changes^19^,^38^. The pattern of expression and even the function of a significant number of genes can be materially altered by the genomic shock accompanying polyploidization^13^. These changes provide a source of variation for natural selection to act upon, with the result that polyploidization has been, and continues to be, a major driver of evolution in the flowering plants^39^. For example, genes involved in energy, metabolism, cellular biogenesis and plant hormonal regulation are up regulated in some allotetraploids, frequently within one or a few generations, and can result in an altered phenotype and ecology^38^,^40^,^41^,^42^. The process could in principle result in the loss of stability at the polyploid level of a reference gene which operates well at the diploid level, although this is unlikely for genes which act in basal processes such as glycolytic pathway (eg. *PGK*)**,** structure (eg. *ACTIN*) and normal physiological function (eg. *EF-1α*). However, the exact regulatory mechanisms underlying these reference genes in polyploids are still poorly characterized. Therefore, determining the transcriptional regulatory mechanisms will be the next important step.

## Methods

### Plant materials and growth conditions

Plants of diploid (2*x*) and tetraploid (4*x*) *C. nankingense*, and 4*x* and hexaploid (6*x*) *C. zawadskii* were obtained from the Chrysanthemum Germplasm Resource Preserving Centre, Nanjing Agricultural University, China. (32°05′N, 118°8′E, 58 m altitude). All plants were propagated by cuttings and grown in a greenhouse held at 22°C during the day and at a minimum of 15°C during the night. The relative humidity varied from 70 to 75% and no artificial light was given.

### RNA isolation and cDNA synthesis

Harvesting of leaves (the third and fourth true leaves from the shoot apex) was carried out in the morning of a sunny day (April 4, 2014, 10:00). Within a maximum of 5 min, the excised leaves were snap-frozen in liquid nitrogen and stored at −80°C until required. Total RNA was extracted using the TRIzol reagent (Takara, Japan). Before reverse transcription, total RNA was treated with RNase-free DNase I (Takara) at 37°C for 30 min to remove any contaminating genomic DNA. The integrity of the RNA preparations was assessed by agarose gel electrophoresis and the concentration of each sample measured using a NanoDrop ND-1000 spectrophotometer (NanoDrop Technologies, USA). Only samples showing an A_260_/A_280_ ratio of 1.9–2.0 and an A_260_/A_230_ ratio >2.0 were used for further analysis. To compare relative transcript abundances estimated from both qPCR and RNA-Seq, total RNA extracted from 2*x* *C. nankingense* was subjected to the RNA-Seq procedure, using an Illumina (San Diego, CA, USA) HiSeqTM 2000 device, following the manufacturer's protocols. Briefly, mRNA was purified from total RNA by the addition of beads coated with oligo (dT). A fragmentation buffer was added to generate fragments in the size range 100–400 nt, and these served as the template for the synthesis of the cDNA first strand, achieved by random hexamer priming. The cDNA second strand was produced using a SuperScript Double-Stranded cDNA Synthesis kit (Invitrogen, USA), purified by passing through a QiaQuick PCR extraction kit (Qiagen, Germany), The products were ligated with one another via the incorporation of sequencing adapters, and after agarose gel electrophoresis, a suitable size range of fragments was selected. The resulting library was pair-end sequenced, and the subsequent transcriptome assembly was achieved using the Trinity software package^17^. The qRT-PCR products (exactly) matching each of the ten candidate reference genes were identified using the local Blast module of BioEdit v7.0.9.0. Transcript abundances were obtained by a count of the RPKM^25^.

For qPCR experiments, the cDNA first strand was synthesized from the total RNA preparations described above. A 1 μg aliquot of total RNA was treated with ultraclean Oligo(dT)_18_ and SuperScript III Reverse Transcriptase (Takara), following the manufacturer's protocol. To confirm the absence of contaminating genomic DNA, the cDNA was amplified using primers recognizing a segment of *Chrysanthemum* flowering locus T-like protein (*FT*) gene-specific promoter (Forward primer sequence: 5′-AGCTGTAGCGTAGCGCAAGT–3′, Reverse primer: 5′-CAAACGCAATAACAAGAGCA–3′, GenBank: KF752603.1, PCR product size: 171 bp).

### PCR primers and qPCR

Ten commonly used reference genes were selected (Table 1), based on their performance across development and under different photoperiod treatments in either *C. lavandulifolium*^4^ or *C. nankingense*^17^. Primers were designed and optimized using a standard set of design criteria (e.g., target 3′-untranslated region for primer design, no hairpin/dimer/false priming/cross dimer structures, primer Tm = 55–60°C, length 18 to 25 bases and GC content between 40 and 60%), which generate a unique and short PCR product (between 80 and 250 bp) of the expected length to facilitate multiparallel qPCR using a standard PCR program.

The qPCR design, calculations and statistics used followed the MIQE guidelines^36^,^37^. Each 20 μL PCR contained 5 μL diluted cDNA, 10 μL 2 × SYBR® Premix Ex TaqTM II (Takara), 0.6 μL of each primer (10 μM) and 3.8 μL ddH_2_O. Primers and qPCR reagents were mixed and a standard volume was aliquoted into each reaction of MicroAmp optical 8-tube strip (0.2 ml) to minimize the number of pipetting steps. To minimize the risk of contamination, all PCR operation performed in a clean environment free of dust under a positive airflow hood and check for DNA contamination of primer (by PCR reactions on no template (water) controls, NTC)^43^.

The reactions were performed in a Mastercycler ep realplex 2 S device (Eppendorf, Germany). To counteract the possibility of systematic variation across the PCR block, the reactions were fullly randomized within different 8-tube strips. Reactions based on the same primer pair were run together, and one strip per block was used as an inter-run calibrator to assess the plate-to-plate variation^43^. The temperature cycling regime comprised an initial denaturation step (95°C/2 min), followed by 40 cycles of 95°C/30 s, 55–58°C (primer-dependent, see Table 1)/30 s, 72°C/30 s. A melting curve analysis (60–95°C, Table 1) and 10% PAGE gel electrophoresis (19:1 acrylamide: bisacrylamide, 7.5 M urea, 50 mM Tris-borate-EDTA, pH 7.8, fragments visualized by silver staining) was conducted following each assay to confirm amplification specificity. Four biological replicates per taxon were analysed, and each sample was represented by three technical replicates.

### Statistical analysis

The LinRegPCR program^44^ was used to calculate the threshold cycle (Ct) and the PCR efficiency and correlation coefficients for each primer pair. Stability was assessed using both geNorm and NormFinder software. The former calculates a stability value (M) for each gene^8^, with an M value <0.5 being taken as an indicator of stable transcription^45^. The latter provides a direct measure of the variation using an ANOVA-based model and ranks the candidate genes accordingly^9^.

To determine the influence of the choice of reference genes on normalization outcome, the second stable gene in the comparisons 2*x* *vs* 4*x* *C. nankingense*, 4*x* *vs* 6*x* *C. zawadskii* and between all four taxa, was taken as the target, and their transcript abundances normalized using either a stable or an unstable reference gene. RAD51 was known to be a key pathway in cells for the homologous recombination and repair of DNA damage^26^. The transcription of *RAD51* was also quantified using both stable and unstable reference genes (the isolation and primer details provided in Supplementary information). Fold changes in transcript abundance were calculated using the 2^−ΔΔCt^ method^24^.

## Author Contributions

Conceived and designed the experiments: H.W., J.J. and F.Z. Performed the experiments: H.W., F.C. and S.C. Analyzed the data: H.W., J.J. and F.Z. Contributed reagents/materials/analysis tools: H.W. and J.J. Wrote the paper: H.W. J.J. All authors read and approved the final manuscript.

## Supplementary Material

Supplementary Information Reference gene selection for cross-species and cross-ploidy level comparisons in Chrysanthemum spp.

## Figures and Tables

**Table 1:** Primer sequences and amplicon characteristics

GenBank ID	Gene	Primer Sequence (5′–3′)	Annealing Tm(°C)	Amplicon Length (bp)	Melting Tm(°C)	PCR Efficiency	Regression Coefficient
**KF305681**	*EF-1α*	TTTTGGTATCTGGTCCTGGAG CCATTCAAGCGACAGACTCA	58.0	151	82.7	0.9710 ± 0.0101	0.9985 ± 0.0004
**KF305684**	*PP2A*	GCTTTCGTAATCGCTTTTGG ATGGATTCACCTCGATTTGC	57.3	183	80.2	0.8926 ± 0.0132	0.9950 ± 0.0003
**CMF007119**	*GAPDH*	CTGCTTCTTTCAACATCATTCC CTGCTCATAGGTAGCCTTCTTC	58.0	170	86.0	0.9812 ± 0.0122	0.9950 ± 0.0003
**KF305683**	*ACTIN*	AGCTTGCATATGTTGCTCTTGA TTACCGTAAAGGTCCTTCCTGA	58.7	244	83.4	0.8974 ± 0.0121	0.9922 ± 0.0004
**KJ524574**	*MTP*	GGTGTTATGATTGGTGCTGCT ATCTATCTCTCGTGGGGTGC	59.0	162	80.7	0.9906 ± 0.0113	0.9951 ± 0.0003
**KJ524573**	*SKIP16*	AAAAGTTTGTGTGGGCTGATTG TGATAGTAGAAGAAGCCGCCA	58.8	163	81.2	0.9523 ± 0.0110	0.9985 ± 0.0004
**KJ524576**	*PGK*	ATACCTAATGGACGAAGAGAACAA TTATTGTCGTCTTTACTACCAGCA	57.7	225	81.6	0.9812 ± 0.0122	0.9922 ± 0.0004
**KJ524577**	*TIP41*	CTGAAAAGTTGGCTGGTAATGAG AAAGAATAGATCGAAAAACGGTGAT	57.7	177	84.8	0.9465 ± 0.0092	0.9967 ± 0.0003
**KF305685**	*TUB*	CTCGAACGCATCAACGTCTA CGAATCAATAAGCTCGGCTC	57.9	216	82.8	0.9952 ± 0.0092	0.9980 ± 0.0003
**KJ524575**	*PSAA*	GTGGATTTCTCATAGTTGGTGCT AGATCGTTGTATCGAGTAGTTGGA	57.9	85	79.6	0.9952 ± 0.0092	0.9989 ± 0.0004
