# Supplementary material for: Reference gene selection for cross-species and cross-ploidy level comparisons in Chrysanthemum spp
Source: Sci Rep. 2015 Jan 28;5:8094. doi: 10.1038/srep08094 (PMC4308696; doi:10.1038/srep08094)
Supplement: Supplementary Information — Reference gene selection for cross-species and cross-ploidy level comparisons in Chrysanthemum spp. [file srep08094-s1.pdf]

Title: Reference gene selection for cross-species and cross-ploidy level comparisons in *Chrysanthemum* spp.

Authors: Haibin Wang[1, 2], Sumei Chen[1], Jiafu Jiang[1], Fei Zhang[1], Fadi Chen*[, 1, 2].

The isolation of CnRAD51

Plants of tetraploid *C. nankingense* were obtained from the Chrysanthemum Germplasm Resource Preserving Centre, Nanjing Agricultural University, China (32°05′N, 118°8′E, 58 m altitude). The third and fourth true leaves from the shoot apex were snap frozen in liquid nitrogen and stored at -80ºC until required. Total RNA was extracted using the TRIzol reagent (Takara, Japan). Before reverse transcription, total RNA was treated with RNase-free DNase I (Takara) at 37°C for 30 min to remove any genomic DNA contamination. The integrity of the RNA preparations was assessed by agarose gel electrophoresis and the concentration of each sample was measured using a NanoDrop ND-1000 spectrophotometer (NanoDrop Technologies). Only the high-quality RNA samples with a measured A260/280 ratio of between 1.9 and 2.0 and an A260/230 ratio >2.0 in a buffer at neutral pH were used for further analysis. The cDNA first strand was synthesized from a 1 μg aliquot of total RNA using ultraclean Oligo(dT)$_{18}$ and SuperScript III Reverse Transcriptase (Takara), according to the manufacturer’s instructions to maximize cDNA length and yield.

The subsequent PCR used degenerate primer pairs (three forward DP primers and three reverse DP primers, sequences given in Supplementary Table 1) which targeted *RAD51*, designed from an alignment of the *RAD51* polypeptides of *Populus nigra* (BAF02935.1), *Cucumis sativus* (XP_004144057.1), *Phoenix dactylifera* (XP_008783310.1), *Citrus sinensis* (XP_006474560.1), *Zea mays* (NP_001104919.1), *Solanum lycopersicum* (NP_001233788.1), *Vitis vinifera*

(XP_002273803.1), *Oryza sativa* (BAB85491.1), *Nicotiana sylvestris* (XP_009789565.1), *Triticum aestivum* (ACH42252.1), *Brassica rapa* (XP_009131820.1), *Medicago truncatula* (XP_003609937.1), *Arabidopsis thaliana* (BAE99388.1). For the 3′ RACE, the first cDNA strand was synthesized using an oligo (dT) primer incorporating the sequence of the adaptor primer, followed by a nested PCR using the gene-specific primer pair GSP3-1/3-2/3-3 and the adaptor primer (sequences given in Supplementary Table 1). For the 5′ RACE, the nested PCR used the 5′ RACE adaptor primer (Abridged Anchor Primer, AAP), the Abridged Universal Amplification Primer (AUAP) provided with the 5′ RACE System kit v2.0 (Takara) and the internal gene-specific primer pair (GSP5-1/5-2/5-3, sequences given in Supplementary Table 1). The gene's open reading frame (ORF) was identified using www.ncbi.nlm.nih.gov/gorf/gorf.html and amplified using primers Full-F/Full-R (sequences given in Supplementary Table 1). The qRT-PCR primers (qCnRAD51-F/R) were also shown Supplementary Table 1. Each PCR amplicon was cloned using a PMD19 TA cloning kit (Takara) and sequenced for verification. A multiple alignment of the predicted gene product with homologs was carried out using DNAMAN software v5.2.2 (Lynnon Biosoft, Canada), and a subsequent phylogenetic analysis was carried out using MEGA 5.0 software (http://www.megasoftware.net/mega.php).

The full length *CnRAD51* cDNA was a 1,363 bp sequence, comprising a 1,038 bp ORF, a 99 bp 5′-UTR and a 226 bp 3′-UTR. The sequence showed significant homology to other plant *RAD51* genes. At the peptide level, the level of identity was 91.8%, reaching > 95% in the most conserved regions (Supplementary Fig. S1). The phylogenetic analysis showed that the most closely related sequences to *RAD51* were the homologs from *Populus nigra* and *Cucumis sativus* (Supplementary Fig. S2). The total results suggested that the RAD51 we obtained could be used in the further

study.

Supplementary Information

Supplementary Table 1. PCR primer sequences used for *CnRAD51* cloning and qRT-PCR.

Supplementary Fig. S1. The multiple alignment of the CnRAD51 with homologs.

Supplementary Fig. S2. The Phylogeny of CnRAD51 proteins.

Table 1 PCR primer sequences used for *CnRAD51* cloning and qRT-PCR

Primers	Sequence (5'–3')
**DP-F1**	GCACGGCCCCTTCCCNRTNGARCA
**DP-F2**	GGAAGGACCTGCTGCAGATHAARGGNAT
**DP-F3**	CCCAGCGGCTGGAGATHATHCARNT
**DP-R1**	GGAGGAGATCACCTTGCAGATNCKYTCYTC
**DP-R2**	GGTGGCGTGGGCCATDATRTT
**DP-R3**	CGGGTCTCGACCATCATGSWNGCNGCYTC
**dT-AP**	AAGCAGTGGTATCAACGCAGAGTACTTTTTTTTTTTTTTTT
**AP**	AAGCAGTGGTATCAACGCAGAGTAC
**GSP3-1**	GAGGTGAAGGGAAGGCGATGT
**GSP3-2**	TGGATTGAATGGTCCTGATGT
**GSP3-3**	AACGGAAGTTGACAAGTTACAC
**GSP5-1**	GGTTTCGACCATCATTGAAGCAG
**GSP5-2**	TCCAAAACATCAGGACCATTCA
**GSP5-3**	GGAGGTGAAGGGAAGGACCT
**Full-F**	CTCTTCTTCACCATTTTCCTTCA
**Full-R**	CTCTCTTCCTTTCTGATGCGTTTT
**qCnRAD51-F**	TTCTGGGAGAGGTGAGCTGT
**qCnRAD51-R**	GATTTGAGGCCCAGAAAACA

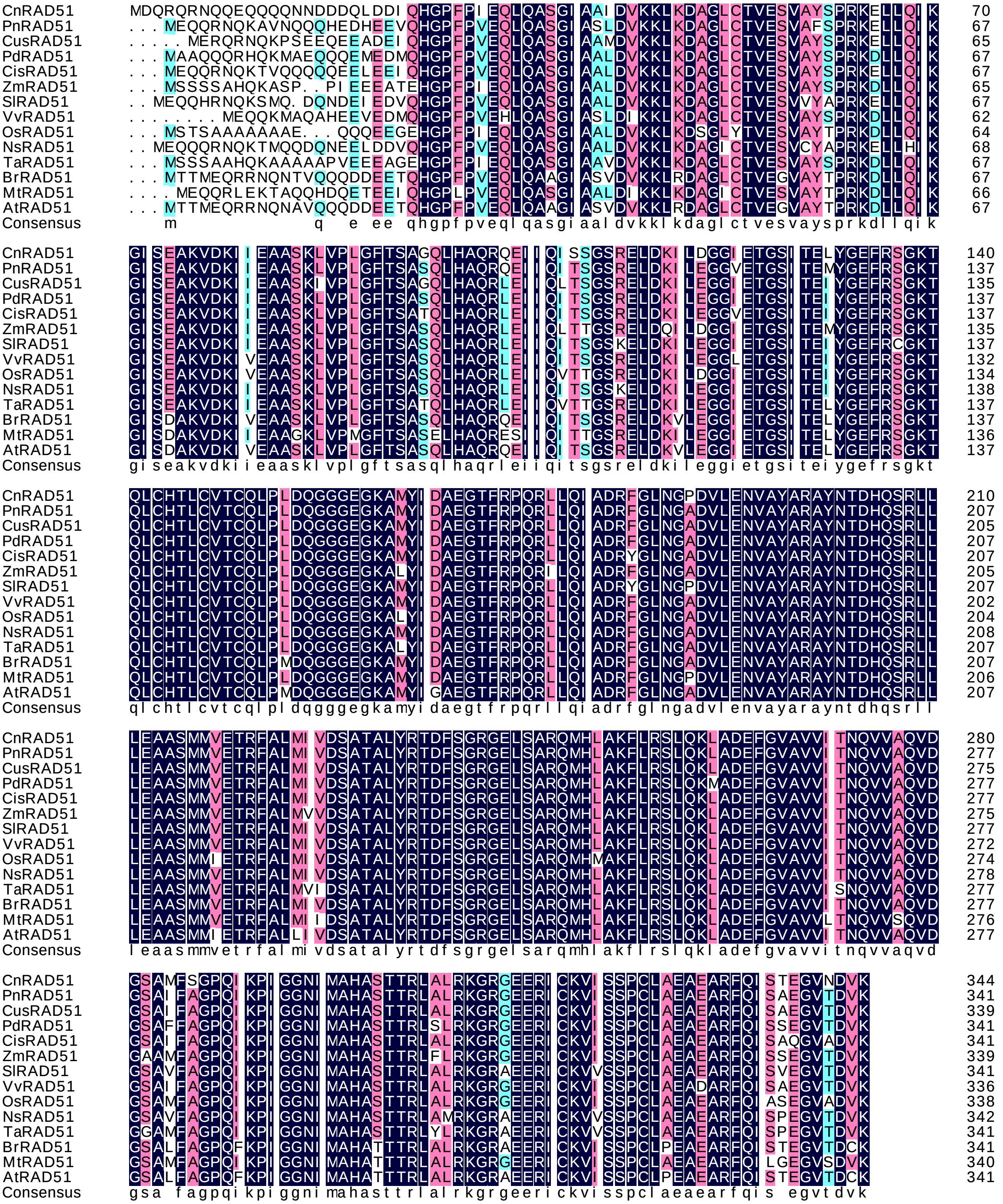

Supplementary Fig. S1. The multiple alignment of the CnRAD51 with homologs: *Populus nigra* PnRAD51 (BAF02935.1), *Cucumis sativus* CusRAD51 (XP_004144057.1), *Phoenix dactylifera* PdRAD51 (XP_008783310.1), *Citrus sinensis* CisRAD51 (XP_006474560.1), *Zea mays* ZmRAD51

(NP_001104919.1), *Solanum lycopersicum* SlRAD51 (NP_001233788.1), *Vitis vinifera* VvRAD51 (XP_002273803.1), *Oryza sativa* OsRAD51 (BAB85491.1), *Nicotiana sylvestris* NsRAD51 (XP_009789565.1), *Triticum aestivum* TaRAD51 (ACH42252.1), *Brassica rapa* BrRAD51 (XP_009131820.1), *Medicago truncatula* MtRAD51 (XP_003609937.1), *Arabidopsis thaliana* AtRAD51 (BAE99388.1).

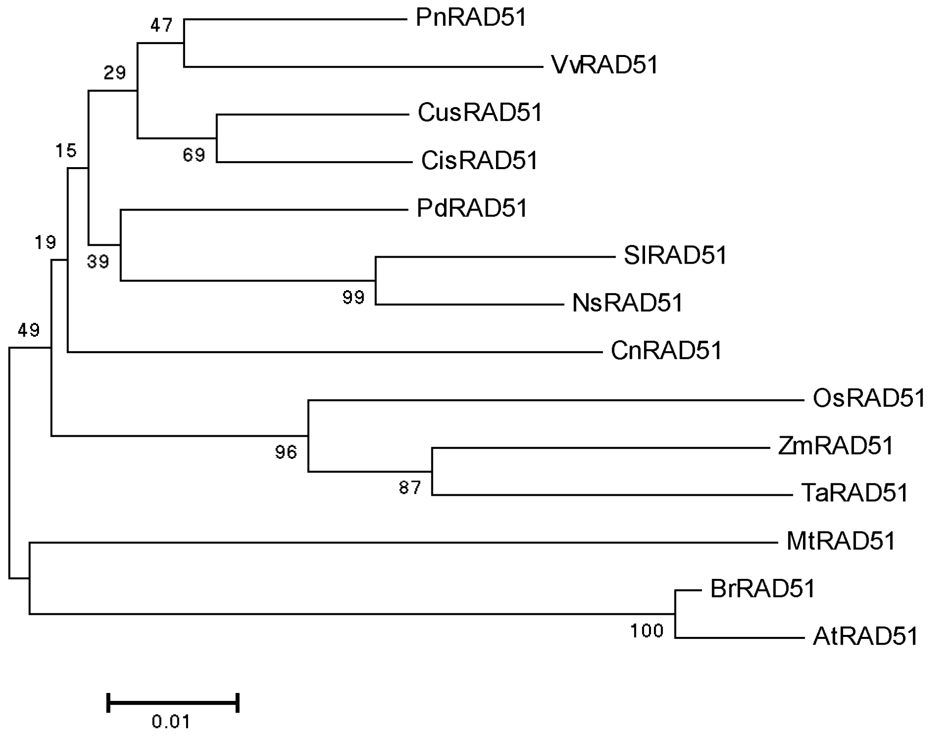

Supplementary Fig. S2. The Phylogeny of CnRAD51 proteins: *Populus nigra* PnRAD51 (BAF02935.1), *Cucumis sativus* CusRAD51 (XP_004144057.1), *Phoenix dactylifera* PdRAD51 (XP_008783310.1), *Citrus sinensis* CisRAD51 (XP_006474560.1), *Zea mays* ZmRAD51 (NP_001104919.1), *Solanum lycopersicum* SlRAD51 (NP_001233788.1), *Vitis vinifera* VvRAD51 (XP_002273803.1), *Oryza sativa* OsRAD51 (BAB85491.1), *Nicotiana sylvestris* NsRAD51 (XP_009789565.1), *Triticum aestivum* TaRAD51 (ACH42252.1), *Brassica rapa* BrRAD51 (XP_009131820.1), *Medicago truncatula* MtRAD51 (XP_003609937.1), *Arabidopsis thaliana* AtRAD51 (BAE99388.1)
